# Mycetoma-like cutaneous leishmaniasis

**DOI:** 10.1590/0037-8682-0420-2024

**Published:** 2025-05-09

**Authors:** Beatriz Zimermano Coimbra, Ana Cláudia Cavalcante Espósito, Murilo de Oliveira Lima Carapeba, Marilda Aparecida Milanez Morgado de Abreu

**Affiliations:** 1 Universidade do Oeste Paulista, Programa de Pós-Graduação Stricto Sensu em Ciências da Saúde, Presidente Prudente, SP, Brasil.; 2 Hospital Regional de Presidente Prudente, Departamento de Dermatologia, Presidente Prudente, SP, Brasil.; 3 Universidade do Oeste Paulista, Faculdade de Medicina, Presidente Prudente, SP, Brasil.

**FIGURE 1:**
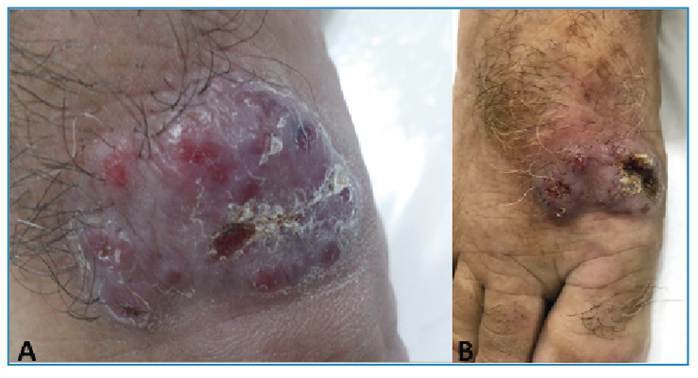
Dorsum of the right foot: **A:** Inflammatory nodosity (mycetoma-like aspect); **B:** Verrucous plaque after 2 years of evolution.

**FIGURE 2:**
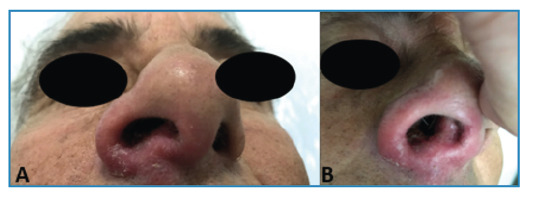
**A and B:** Erythematous infiltrative lesion in the right nostril.

Cutaneous leishmaniasis is a protozoan disease caused by the bite of a sandfly that injects parasites into its host, with *Leishmania (Viannia) braziliensis* being Brazil's most common etiological agent^1^. Most patients manifest classical symptoms. However, there are atypical forms of this disease^2^ ^,^ ^3^. We report the case of a 53-year-old Brazilian man presenting with an inflammatory nodosity on the dorsum of the right foot causing pain, with local fluctuation and bloody discharge for 6 months (Figure 1A). Abscess and mycetoma hypotheses were raised and ciprofloxacin 500 mg was prescribed every 12 h for 14 days. One month later, the patient showed little improvement, and a biopsy of the lesion was performed. The histopathological examination was compatible with chronic abscessing dermatitis. Sulfamethoxazole and trimethoprim were prescribed; however, the patient was lost to follow-up. Two years later, the patient returned with erythema and infiltration in the right nostril (Figure 2A and B ), and the lesion on the right foot persisted (Figure 1B). The diagnostic hypothesis was mucocutaneous leishmaniasis. Histopathological examination of a new biopsy specimen from the foot lesion revealed superficial deep granulomatous dermatitis. Despite normal laboratory test results and negative results for acid-fast bacilli, leishmania, and fungi in direct investigations, the diagnosis was confirmed using a positive indirect immunofluorescence test for leishmaniasis. Few reports of mycetoma-like leishmaniasis, as observed in this case, have been published in the literature. Glucantime was prescribed at two ampoules per day for 30 days, achieving total lesion regression without recurrence at the seven-month follow-up.
